# Requiring code sharing to strengthen transparency and trust in research

**DOI:** 10.1371/journal.pmed.1005107

**Published:** 2026-05-26

**Authors:** Helen Lumbard, Lauren Cadwallader, Devin Soper, on behalf of the PLOS Medicine Staff Editors

**Affiliations:** 1 Public Library of Science, Cambridge, United Kingdom; 2 Public Library of Science, San Francisco, California, United States of America

## Abstract

Recognizing that code is as essential a research artifact as the data it analyzes, this Editorial outlines how PLOS Medicine is strengthening its code sharing policy to further ensure reproducibility and trust in the scientific record.

## Teaser

*PLOS Medicine* has always championed open science and data transparency. Now, recognizing that code is as essential a research artifact as the data it analyzes, we are strengthening our code sharing policy to further ensure reproducibility and trust in the scientific record.

At *PLOS Medicine*, our mission has always been to publish research that not only advances human health but also embodies the highest standards of openness, trustworthiness, and scientific rigor. Open science is not an abstract ideal—it is foundational to medicine itself. From the design of clinical trials to the interpretation of complex epidemiological models, transparency in every aspect of the research process is essential if science is to serve clinicians, patients, policymakers, and the public with integrity and impact. *PLOS Medicine* has championed open and transparent practices for over 20 years, for example, requiring data sharing, integrating with medRxiv to facilitate preprint posting [1], and adopting new policies to strengthen standards for rigorous research reporting [2–4].

Today, we are proud to announce the rollout of our code-sharing policy—a concrete step toward strengthening reproducibility and accountability in medical research by ensuring that the code underlying published findings is shared openly and responsibly.

## Why code sharing matters in medicine

Modern medical science increasingly depends on computational approaches: from algorithms that process large genomic datasets, to statistical scripts underpinning observational studies, and code that implements predictive models of disease progression. These digital tools are not peripheral - they are central to how conclusions are reached, interventions are judged, and evidence influences practice. Yet, historically, much of this code has remained invisible to readers, reviewers, and secondary analysts.

By making author-generated code accessible at the time of publication, *PLOS Medicine* is strengthening the scientific record in three key ways:

1. Reproducibility—code sharing allows independent researchers to reconstruct analyses, verify findings, and explore alternative interpretations with confidence.
2. Usability—shared code becomes a usable resource for others to build upon, adapt, and extend to new questions that matter to patients and societies.
3. Trust—transparency fosters trust among clinicians, funders, and the public by making every step of a research finding accessible and open to scrutiny.

Code sharing is not simply an add-on to publication; it is part of robust scientific communication.

## What the policy means for authors

Under the new policy, authors submitting articles to *PLOS Medicine* in which author-generated code supports the results of the manuscript are expected to make that code publicly available upon publication. Code should be accompanied by sufficient documentation to enable reuse and, wherever possible, deposited in repositories that provide persistent identifiers (see Box 1). This expectation mirrors our long-standing commitment to open data and open materials, and reflects the reality that code is as essential a research artifact as the data it analyzes.

**Box 1. Navigating the *PLOS Medicine* code-sharing policy**

**What?**

All author-generated code required to reproduce the findings, analysis, and visualizations reported in the manuscript, as well as related documentation. Please include a README file describing the code in an accessible manner, the environment in which it should be run, and any existing dependencies.

**Where?**

Where code has been shared in a non-permanent repository, such as Github or Bitbucket, a snapshot of the code should be archived in a permanent, public repository that issues persistent identifiers, for example, Zenodo, Code Ocean, or the Software Heritage archive.

**When?**

Code will need to be shared before publication, and we will request that it is made publicly available during our pre-acceptance checks. Editors and reviewers may request to see your code, especially if it is central to the claims of the study.

For more detailed information, please see the full code availability policy and accompanying code-sharing guidance.

We recognize that legitimate ethical or legal considerations, for example, patient privacy limitations or contractual restrictions, may sometimes constrain what can be shared. In such cases, authors should be transparent about these constraints and describe how code can be accessed or evaluated under appropriate controls. Editorial and peer review will continue to evaluate compliance thoughtfully and fairly, with the shared goal of maximizing openness without compromising ethical obligations.

## A cultural shift, not just a policy

Implementing this policy is not merely a procedural change—it signals a transformation in how medical research communicates and collaborates. Medicine tackles complex human challenges where every decision can have profound consequences. The computational tools and analyses that underpin these decisions should be visible, interpretable, and reusable, not confined to private scripts or inaccessible silos. By embracing open code, *PLOS Medicine* is committing to a future where clinical and translational research is not only open to readers but open to re-use, scrutiny, and community innovation.

We are grateful to the many authors, reviewers, and editorial board members who have championed open methods and supported us with developing this policy. Many in our community already share code generously, with 53% of articles published in 2025 sharing code [5]; this policy formalizes that culture and encourages broader adoption of best practices. We will continue to provide guidance and support to authors as they navigate these changes, and we look forward to learning from our community’s experience as this policy takes effect.

Our commitment to code sharing is a milestone in a larger journey toward fully open and accountable science. Over the course of 2026, we will run a soft rollout, offering support and learning opportunities for authors, before making code sharing mandatory in 2027. We will continue to listen, learn, and refine our approach and support authors, ensuring that the policy strengthens reproducibility, fosters innovation, and accelerates scientific discovery.
